# Meta-analysis: a tool for constructing theories or evaluating interventions or simply proving everyday assumptions?

**DOI:** 10.3389/fpsyg.2024.1377336

**Published:** 2024-10-24

**Authors:** Erich H. Witte, Ivo Ponocny

**Affiliations:** ^1^Psychological Institute, University of Hamburg, Hamburg, Germany; ^2^Faculty of Psychology, Sigmund Freud University Vienna, Vienna, Austria

**Keywords:** meta-analysis, theory testing, reliability, validity, error theory, abduction

## Abstract

After a brief consideration of the development of meta-analyses as a joint discussion of results from a research area across development stages 0, 1, 2, it is concluded that the present form 2.0 is unsuitable to serve as a basis for theory building. Further development of this tool into a meta-analysis 3.0 is necessary for this purpose which requires the validity of the independent variables in the primary studies, the reduction of the error variance of the dependent variables, a stability of the effects over the primary studies and a quantitative comparison between observed and predicted effects in the primary studies. In the current meta-analyses 2.0, a concrete single-case approach creates the impression that mainly everyday ideas are investigated, which one would like to generalize to a population of other conditions. Furthermore, the results of the existing meta-analyses are either homogeneous and very small or heterogeneous. Meta-analysis 2.0 searches for the instability of the measurements under a specific topic with methods of induction. The procedure of a meta-analysis 3.0 is described in general and carried out hypothetically and with an empirical example. It searches for the stability of quantitative reconstructions of data over different topics with the method of abduction. The conclusion can be summarized as that meta-analysis 3.0 is indispensable as a tool for theorizing, and theorizing presupposes meta-analysis 3.0. The link between this interdependence is abduction in contrast to induction as a research strategy.

## 1 Introduction

Is there anything more to say about meta-analysis? It does not seem so, but reading two current relevant articles we find the following citations:

1. “The key idea is meta-analytic thinking: Appreciate any study as part of a future meta-analysis. With good understanding of meta-analysis, we know how essential it is that our research literature be complete and trustworthy, and that all studies be reported in full and accurate detail “(Cumming, 2014, 27). The author considers this as the new statistics which will avoid all the past deficits.

2. “Nevertheless, meta-analytic thinking not only fails to solve the problems of *p*-hacking, reporting errors, and fraud, it dramatically exacerbates them. In our view, meta-analytic thinking would make the false-positives problem worse, not better” (Nelson et al., 2018, 527f). Should we stop with meta-analytical thinking or force its application in the future? There is a high intensity of publishing meta-analyses, absorbing research time to be invested into this activity. Recently, new meta-analytical activity takes place in order to replicate an effect by preregistered studies, avoiding a publication bias in the sense articles tend to present only significant results.

We will not discuss meta-analytical methods or sophisticated methodological problems^1^ in technical detail, we concentrate on the tasks this approach should undertake and use as an example the most common approach with two groups, a control group and an experimental group, or a control group and a treatment group in the applied field. What are the central tasks of the meta-analyses?

There are several phases in the emergence of meta-analysis as a technique which we call meta-analyses 0.0 to 2.0. Originally, one reviewed and described empirical results from a field of research. Thus, *meta-analysis 0.0* was solely about the qualitative description of empirical results about a field (Pratt et al., 1940). Perhaps one still reported the number of significant results, but there was no quantitative description of an aggregate empirical result. In the next generation of *Meta-analyses 1.0*, the dominant goal was to compare the effects of psychotherapies (Glass et al., 1981) or the quality of personnel selection using test procedures (Hunter et al., 1982).

A further part was the idea of finding a more complex and therefore a more reliable test of hypotheses by combining significance tests into a global test of a hypothesis. Such idea came up before the term was coined and discussed at the very beginning of statistical tests of significance (Birnbaum, 1954). This idea is still in use (Meta-analysis 2.0) if the mean and the heterogeneity of the means under the combined sample of all studies is considered as a basis for a significance test (Cumming, 2011; Hunter and Schmidt, 1990; Langan et al., 2019).

In the quantitative *review part* (Meta-analysis 1.0), the focal intention is the amount of the effect of an intervention (e.g., psychotherapy) generally and the quantitative differences between variants of psychotherapy. Cohen’s (1977) *d*-value is a popular standardized statistic of the single study using the empirical observation in each study (Baguley, 2009), based on the differences of means *m*_1,2_ and related to the observed standard deviation s: *d* = *m*_1_ – *m*_2_/*s*.

The *statistical part* combines the individual tests of significance into a complex test which seems to be more trustworthy because the test is grounded on more than one study and therefore a kind of replication, which is now the main theme of statistical significance testing (Nosek et al., 2022). The dependent variable of a meta-analysis is such a *d*-value or a correlation coefficient that can be averaged over several studies with the result of a mean *d*-effect size or correlation (Grissom and Kim, 2012). These statistics can now be used for a statistical test under a fixed or random effects model (Meta-analysis 2.0) (Borenstein et al., 2009; Hedges and Olkin, 1985; Hunter and Schmidt, 1990; Lakens, 2013; Rosenthal and DiMatteo, 2001; Rosenthal and Rubin, 1982; Schulze, 2004; Witte et al., 2020; Witte et al., 2022; Witte and Zenker, 2016a; Witte and Zenker, 2016b; Witte and Zenker, 2021).

If we look at the chronological order of these different aims of a meta-analysis, its beginning is the combination of independent tests of significance (Birnbaum, 1954). The test is not to utilize the observed *t*- (the test statistic used) or *d*-values - eliminating the sample sizes in each study – because in the general case it must be assumed that “there is no known or reasonably convenient method available for constructing a single appropriate test of H_0_ based on (*t*_1_, *t*_2_, …, *t_k_*)” (Birnbaum, 1954, 560). Such a test, like Fisher’s formula of combining independent *p*-values, must be based on the probability density function of *t_i_* under H_0_ and not on the observed *t_i_* themselves. Such a complex, integrated test is one central aim of a science which would like to integrate the preliminary knowledge of single studies because scientific knowledge is embedded in the well supported informative theories with corroboration strategies in different labs. We will come back to this problem with an alternative approach (Witte and Zenker, 2017a; Krefeld-Schwalb et al., 2018). Such a combined test is developed using the dependent statistics as data (*d*-values or other statistics) testing against a null hypothesis of the integrated statistics from the studies. Meta-analysis 2.0 is the common way to evaluate treatments, e.g., in education (Hattie, 2008; Lipsey and Wilson, 1993); personnel selection (Schmidt and Hunter, 1998) or health care (Ioannidis, 2016), and regarding scientific misconduct (Fanelli, 2009).

Recent large scale meta-meta-analyses demonstrate that behavioral science publications typically report observed effects that are either small and homogenous or large (r) but heterogeneous, entailing large observed variance (Schauer and Hedges, 2020, p. 39; Olsson-Collentine et al., 2020; Linden and Hönekopp, 2021).

The homogenous share of observations thus implies that such effects are typically overlain by the standard measurement error, hence becoming quasi-*unobservable*. Consequently, the *theoretically postulated experimental effect is hardly supported since* there is a nearly infinite number of potential influences of such small deviations from the zero-effect null hypotheses. This applies, by the way, not only to the average-centered view, but also to a person-centered one, expressing that the effect under consideration affects only a small share of the participants (see also Witte, 2024). If our theories concentrate on the means of the two groups as their basis of explanation and prediction the single subjects should follow the theoretical assumption. Such an accordance between an average-centered with a person-centered approach can only be reached by a massive reduction of the measurement error, because a d-value greater than *d* = 3.3 separates the two distributions in the way that only 5% of the scale values are shared and does not follow the theoretical prediction [see Cohen, 1977, Table 2.2.1 p. 22].

In turn, the heterogenous share of observations implies that such effects can be predicted only under conventionally unacceptably large prediction error-rates. The upshot is that an *empirically adequate* theoretical construct needs to predict a much larger, homogeneous effect than it is observed in the common meta-analytic strategy. Accordingly, the common meta-analysis 2.0 is not a tool to evaluate theoretical generalizations (Witte and Zenker, 2017a; Krefeld-Schwalb et al., 2018), but a tool to evaluate empirical discoveries under a random effects model what has been observed empirically.^2^ This difference is represented in a fixed effect model as a theoretical specification and a random effects model as an inductive generalization (Erez et al., 1996; Hedges, 1983; Hedges and Vevea, 1998; Hunter and Schmidt, 2000). The second approach—application—is evaluated against random and the first approach is evaluated against truth as a comparison level (Witte and Zenker, 2017a; Krefeld-Schwalb et al., 2018).

As a result, many data obtained from meta-analyses do not have a capability of theory construction which would require a concept of *data’s theory construction capability (dtcc)* that is grounded on the following characteristics:

- The data on a scale is valid for a fixed theoretical construct (not modified by a statistical parameter *s* of the observed scale values in a study).
- The error distribution of the dependent variable *d* is small.
- The theoretically predicted difference is large and stable over conditions and time compared to a random hypothesis (the theoretical construct is the intuitive integration of different empirical observations under one concept without fitting the data).
- The theoretically predicted difference is highly like the empirical observation (not only significant but psychometrically similar as predicted).

This concept of *data’s theory construction capability* is the aim of a meta-analysis for theory construction. Obviously, the meta-analysis 2.0 is not able to produce such data (Linden and Hönekopp, 2021) and thus, the empirical ground for a theory construction is missed and the automatic consequence is the lack of theories. There are few empirical results in psychology with such a capability. We must pave the way for a meta-analysis 3.0 to build the base ground for theory construction as a process of abduction to formulate theories from which effects can be deduced.

An alternative approach toward meta-analysis 2.0 for theory or hypotheses testing is provided by Bayesian methods [for an introduction, see Gronau et al. (2021)], which are more dynamic regarding the theory to be investigated than classic meta-analyses, wherefore our criticism does not apply in the same way. Exactly for this reason, however, as well as for the assumption of random effects, they serve a different purpose compared to the proposed meta-analysis 3.0 which aims to derive a value from observation data per abduction which is as constant and generalizable as possible, with the only ambition to *construct* a theory. Hypothesis testing is conducted by other procedures, then, using values derived by meta-analysis 3.0 as input. For this reason, we will not refer to this approach in more detail here. [For more general comments about the role Bayesian methods for theory testing, cf. Witte and Zenker (2017a)].

The scientific procedure of abduction is central to us, but at the same time this term has been discussed less in the philosophy of science. At this point, let us explain its meaning as a scientific strategy. It goes back to Peirce (1960) and is currently being discussed intensively (Magnani, 2023; Magnani and Bertolotti, 2017) because it has been recognized that the intuition of the individual researcher cannot be dispensed with.^3^

The principle of abduction in the Peircean sense [following Barrena and Nubiola (2023)] is to set up a hypothesis because then the—at first sight—surprising observation would not be surprising any more but something normal. The choice of this hypothesis is not on objective conclusion, but involves—in contrast to induction and deduction—some kind of intuition, with some degrees of freedom which hypothesis to choose from a potentially infinite set. However, it still involves observations, for which plausible explanations are sought, ideally a lot of observations. Meta-analysis 3.0 aims to provide a sufficient empirical basis for this, since “Peircean abduction consists precisely […] of rearranging various elements that seem unconnected, thus linking what at first glance may seem disparate and irrelevant. Creative thinking therefore implies a continuation of ideas, proceeding further in the train of thought” (Barrena and Nubiola, 2023, p. 1208).^4^ When we will talk of stable patterns to be sought in the following, we do not suggest to harvest certain parameter values, but to provide the bases for patterns which may then initiate innovative ideas, based on the researchers’ intuition.

To outline the specific meaning of this approach and to distinguish it from the known research strategies, it is best to consider the central research strategies together in Table 1.

**Table 1:** Methodological approaches in the research process.

Scientific approaches	Data to constructs	Constructs to data
**Individual-intuitive**	Abduction (creation of hypotheses)	Conduction (combination of hypotheses)
**General-methodological**	Induction (derivation of hypotheses)	Deduction (proof of hypotheses)

Abduction and its feasibility play a crucial role in the discussions about scientific realism. We aim to contribute to this discussion by proposing meta-analysis 3.0 as a method to reduce the danger of subjective or arbitrariness of scientific assumptions, and above all, the danger of pre-maturity of ideas to be tested. In fact, it may be seen as an implementation of claims like in Wylie (1986, p. 293), requesting pragmatic realists to provide a “systematic account of how abductive inferences […] are formulated in the process of building plausible hypotheses.” Acknowledging that the term “meta-analysis” is currently used for theory evaluation, we would like to extend it by applying its approach of data aggregation not only to theory *evaluation*, but also to theory *construction.*

Within the abduction concept, an individual explanation is valid if it is the unrivaled best possible explanation of a set of known data. It is an intuition of a researcher to explain the set of his data without a formal method used under induction fitting the data. Such an explanation uses theoretical constructs behind the data. The opposite process, conduction, uses constructs (theories) to predict an outcome in a specific complex condition. Constructs are used in an intuitive combination.

If the method of induction is not used with the available data, to which population can one generalize from the sample? It is only possible to generalize from a sample to a population if the population is finite. In this case, the sample must have the specific property of representativeness and be a random sample. However, the population of theories is always infinite, so generalization is never possible. This also applies if the theory restricts the population and the conditions. The population of a theory always remains infinitely large. It follows that it is never possible to generalize from random samples to theoretical populations. Induction cannot solve this problem compared to abduction. The advantages and disadvantages of abduction with its intuitive consideration as a new discovery or individual misinterpretation remain.

## 2 Meta-analysis 3.0 as such a tool for theory construction

We know that because of error fluctuations, we always need to integrate multiple studies to get an error-reduced empirical mapping. Depending on the goal, we can perform a meta-analysis 2.0, but it will only give us evidence of discoveries as deviations from chance. For theory construction, we need other empirical data that are subordinate to the concept of theory construction as a goal. As mentioned above, the prototypical concept can be divided into several steps which are shown in Table 2.

**Table 2:** Comparison of meta-analyses 3.0 and 2.0.

Meta-analysis 3.0	
Main goal	Identifying hidden structures for theory construction
Data collection from different fields of research	Collecting empirical evidence
	First abduction steps about relevant elements
	Finding overarching patterns by quantitative reconstruction of means
Improving the measurements of the theoretical parameters	Quantization of data to get almost true values by coarse graining
Sharing the results	Publishing quantized effect sizes and reconstruction rules. Assessing fit of data to current reconstruction (I_SIM_)
Formulating theories	Explaining the reconstruction (retrodiction). Abduction
Evaluation and modification of theories	Reconstruction of data assuming certain laws
	Further abduction: modifications and refinements
	Iterative continuation of the previous steps
Formulating a final theory	Specifying the conditions and the laws for an average individual
Starting a research program	Differentiating and combining theories

Meta-analysis 2.0	
Starting an every-day research question	Concentrating on a specific topic
Literature review	Electronic collection and classification of studies on a specific topic, search for unpublished results by electronic requests
Selection of studies by quality criteria	Differences of the inclusion between meta-analyses 2.0 from various authors by subjective ratings
Statistical aggregation and evaluation	Standardizing of effect sizes for every object-study by the standard deviation of each study, evaluation of effect sizes (small, medium, large) and their homogeneity over the sample of studies, frequent misinterpretation of the effect size as a difference of the individual measurements (average-to-person bias; cf. 4.3)
Summary of existing studies	Aggregating heterogeneous effect sizes under a random effect model, often a look for incommensurable data in one specific topic (cf. Chapter 3)

At the beginning, the search for stable overarching patterns serves as the backbone for the initial abduction process. In order to generate more precise and therefore workable statements, a technical procedure is proposed which reduces measurement error substantially. This will be of help for deriving statements about patterns common to different studies. As a second tool, a coefficient evaluating the link between theory and data is suggested which will ease the evaluation of the success of the current working hypothesis. Having finished the iterative pattern detection, abduction and evaluation loops, finally an elaborated quantitative theory is derived which may include conditions and diversification and constitute the starting point for a coordinated research endeavor—or a meta-analysis 3.0.

### 2.1 The validity of the intervention

In experimental research beyond discoveries of effect sizes, the focus is on testing a theoretical assumption. To do this, one creates an experimental condition that manipulates a theoretical variable and is intended to produce a theoretically predictable result. Validation of independent variables as well as dependent variables is inadequate in most studies (Flake and Fried, 2020; Flake et al., 2022; Hussey and Hughes, 2020). The cost of making a measurement of a theoretical construct that is as error-free as possible is always great because one must isolate the construct from other influences and make its precise measurement. Meta-analysis 3.0 cannot solve the problem of validation, but it must be considered as a prerequisite for interpreting the meaning of the measurements, because data in general can be doubted in their validity (Witte and Zenker, 2017a). As a general prerequisite for integrated measurements across multiple studies, the validity of the measurements must be clarified, which in turn requires the adoption of theoretical constructs. Meta-analysis 3.0 is interface for the theoretical advancement of psychology as an empirical social science because it serves the needed empirical data.

### 2.2 An error-theory with a concrete measuring instrument

Once the question of independent variable’s validity is settled, we must turn to the reliability of the dependent variables as data. In every experiment, a dependent variable is recorded with a measuring instrument. If we now only consider the classic case of measuring the responses of the test participants on a scale, then the reliability of this scale is an indicator for the measurement error. The larger the measurement error, the less likely it is that a similar result will appear in a replication. Replication of such experiments is therefore subject to a corresponding theory. The *abstract* theory of measurement error (Lord and Novick, 1968) ignores its variation due to differences in the varying reliabilities of the specific measurement instruments. However, if we now know the reliability of the measurement instrument, then we can be more precise about the measurement in the concrete experimental condition. The abstract theory can be specified in its relation between true and observed values on the level of data and not only on the level of statistical correction of correlations based on idealized parameters (see below).

We know that people’s ability to differentiate is very limited and that is why we also use a very limited number of categories (3, 5, 7, 9) in rating scales (Preston and Colman, 2000). Because we draw on multiple items and multiple people, we seem to get continuous gradation. However, this variation of the measured values is partly based on measurement errors, as we can see from the reliability of the scales. The continuous differentiation between the scale values and the ability of subjects to differentiate between stimuli has a threshold, as we know from psychophysics. One consequence was the research about the number of categories on a Likert-scale with a high reliability, because with too many categories the reliability decreases. Thus, the Likert-scale and other forms concentrate on few categories to register a reaction on a single item. Furthermore, there are many random context effects that modify the reaction on scales (Schwartz, 1999). With this limit in mind, we must care about a coarser differentiation of scale values to get reliable and reproducible reactions of subjects and the true position of these subjects on a scale, *their empirical psychological trait value* (Nosek et al., 2022). If one cannot collect stable measured values, then one cannot proceed scientifically. (Also, stable changes are stable measured values, one does not have to use constant values as a basis of the measurement, such as personality characteristics or attitudes ignoring their change, e.g., with age). The instability of the measured values is partially caused by the measurement error, the deviation from true score *T*. When considering the classical measurement error, the following formal relationship applies to measurements by a measuring instrument (Lord and Novick, 1968):

$${s^{2}}_{E}={s^{2}}_{obs}-r_{XX^{\prime }}\cdot {s^{2}}_{obs}\;\text{or}$$

$${s^{2}}_{E}={s^{2}}_{obs}\;\left(1-r_{XX^{\prime }}\right)\;\text{with}$$

$$r_{XX^{\prime }}={s^{2}}_{T}/{s^{2}}_{obs}$$

**Figure 1:**
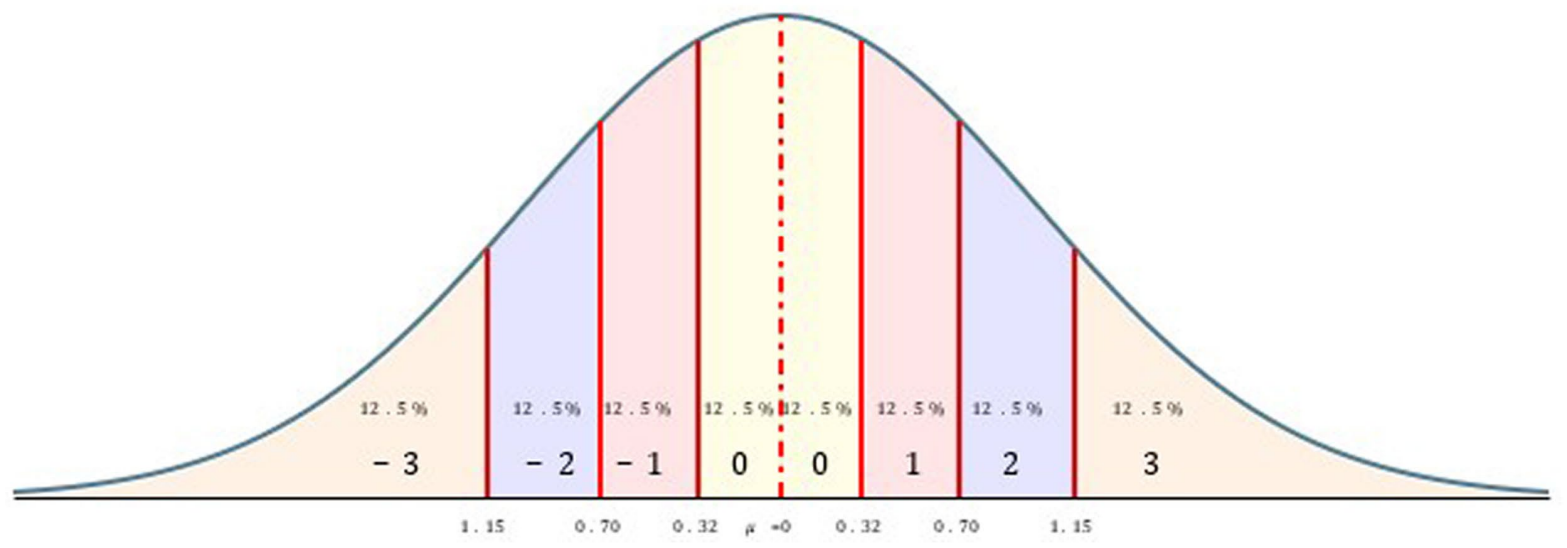
Decomposition of continuous measured values into 7 quanta.

The demand is now usually that in order to reduce the measurement error, one should increase the reliability of the measurement instrument. This reduces the measurement error √s^2^_E_, because the observed variance s^2^_obs_ is then reduced more strongly. But now, if one cannot improve the measurement instrument due to the limited ability of subjects, but still wants to reduce the measurement error, then one can reduce the variance of the observations s^2^_obs_, as can be seen above. Also, in this case the measurement error is reduced. So, if one measures with less differentiation, then these measurements remain more stable. The usual continuous differentiation between the measured values, which is captured by the variance, contains much error fluctuations. By combining continuous measured values into quanta, which then receive a common measured value, one can reduce the error fluctuations and thus increase stability. The reduction of the measured values to coarser quantizations does not represent a loss of information, if the finer differentiation is strongly affected by the error influence. Thus, one is faced with the measurement problem of reducing the observed measurements on a scale with a given reliability into measurement of quanta in such a way that the quantized measurements have lower errors while allowing as much differentiation as is possible, but not more (Witte, 2023, 2024). It will not be possible to eliminate sampling error, i.e., the influence of the mean in a sample on the distribution of measured values, but the mean represents a particularly stable measurement that can be assumed as a reference point in the transformation into quanta. So now, one will want to summarize the measurement values on a scale with a certain reliability in such a way that these values summarized in quanta represent nearly true differences (Witte and Stanciu, 2023). This means that the measurement error is reduced by summarizing outcomes into quantized intervals, establishing a common measurement value for all continuous values in this interval. Under this kind of transformation, the quantized scores are a reasonable approximation of the true scores. We can determine this susceptibility to error as the reliability of the measurement instrument, and we can assume that reliabilities higher than the usual ones between r_XX′_ = 0.50 and r_XX′_ = 0.95 can only be achieved in very rare cases. This range probably covers the anthropometric reliability due to the ability of human beings (Hussey and Hughes, 2020; Wetzel and Roberts, 2020). Combined with this reliability range, one can assign a measurement error range from s_E_ = 0.70 to s_E_ = 0.22, the square root of the reliability. If one now assumes that the continuous measurements on a scale originate from a normal distribution (justified by the Central Limit Theorem), which consists of a combination of an observed value x_i_ associated with an error fraction (s_E_ · x_i_) that increases with the deviation from the mean, then one must construct quanta in such a way that they take into account this greater susceptibility to error. As a theory of measurement, one can now introduce the standard error of measurement as a quantum width, so that the deviation of the continuous measured values from the mean in the range of s_E_ are contracted into a common measured value, so that m_obs_ ± s_E_ = m_quant_ is set as the measured value for such an interval around the mean.^5^ The question now is how to further differentiate for measured values that lie beyond this interval. The idea is not to keep the measurement *distances* for the next intervals constant in s_E_ units, but to keep the *number of* possible measured values in the following intervals constant (subjects in a state of maximal entropy).^6^ But since more extreme values become rarer in a normal distribution, the interval widths must increase in order to assign an equal number of subjects to such an interval. Thus, each interval covers the same number of measured values and the total population of measured values is decomposed into quanta with the same frequency of occupation in the quanta at an uninfluenced starting distribution on a theoretical construct. The deviation of the quanta from the mean can then be easily measured with integers describing the distance from the mean of the sample *m* (see Figure 1 as an example).

Thus, the measurement error of the measuring instrument s_E_ or the reliability as a unit of measurement determines the percentage of continuous measurements that fall within a measurement interval. One decomposes the continuous, normally distributed z-or t-values into intervals so that the percentage of the measured values of m + s_E_ and m-s_E_ remains constant even for the more extreme intervals (with interval widths a, b, …):

$$P\;\left(+\right)=\int_{0}^{sE}z\;dz=\int_{sE}^{sE+a}z\;dz=\int_{sE+a}^{sE+b}z\;dz=\ldots =\text{const}$$

$$P\;\left(-\right)=\int_{-sE}^{0}z\;dz=\int_{-\left(sE+a\right)}^{-sE}z\;dz=\int_{-\left(sE+b\right)}^{-\left(sE+a\right)}z\;dz=\ldots =\text{const}$$

Thus, all intervals theoretically cover the same percentage of measured values like a neutral state before any concrete measurement. As reliability increases, the percentages of measured values that fall within an exterior interval decrease (see Table 3). With this quantization of continuous measured values, the error fluctuations are shifted to the intervals and only if the deviation from the mean is larger than the quantization dependent on the error fluctuation, the measured value is assigned to the next interval. As can be seen from Table 3, this type of measurement becomes coarse. However, this coarsening of the measured values is based on the quality of the measuring instruments due to the limited ability of subjects, which do not allow a stronger differentiation. An anthropometric measurement model cannot avoid quantizations because there are error ranges that cannot be eliminated. Humans are only very coarse measurement carriers, and this property should be considered in a quantization. Then one can assign a high stability to the measured values and use them as starting points for the formation of theories and the replication of theoretical effect sizes. Under this kind of transformation, the quantized scores are a reasonable approximation of the true scores. Simulations of 10,000 samples with sizes of N = 30 and N = 50 from a t-distribution with m = 0 and s = 1 leads to a reduction of the standard deviation from around about s = 1 to about s = 0.04 under different quanta (see Table 4) with the almost constant mean of m = 0. The difference between means is now measured on a scale of quanta with the assumption being a mean difference of true values (Witte, 2023) (see below).

**Table 3:** The relationship between measurement error, reliability, category number and percentage of measured values in the measurement interval.

Measurement error s_E_	Reliability r_XX’_	Category number (quanta)	Percentage of measured values in the measuring interval
0.70	0.50	3	25%
0.45	0.80	5	17%
0.32	0.90	7	12.5%
0.22	0.95	11	8.3%
0.17	0.97	13	7.2%
0.10	0.99	25	3.85%

**Table 4:** The comparison between the continuous measurements (Cont) and the quantized measurements due to the means (m) and standard deviations (s) in two sample sizes *n* = 30 and *n* = 50 and four quantizations.

Quanta	Cont m	Cont s	Quantum m	Quantum s	Cont m	Cont s	Quantum m	Quantum s
3	0.00	1.03	0.5	0.02	0.01	1.04	0.00	0.04
5	0.00	1.08	0.0	0.03	0.01	1.04	0.00	0.03
7	0.00	1.08	0.5	0.03	0.01	1.04	0.00	0.04
11	0.00	1.08	0.5	0.06	0.01	1.04	0.00	0.06
n	30	30	30	30	50	50	50	50

### 2.3 The theoretically predicted difference is stable over conditions and time

Now, if the mean values are the focus, then an experiment should show a clear distinction between the mean values of the control group and the experimental group. Only in this case it can be assumed that the differences are due to the experimental manipulation. Also, only the mean values correspond to theory and should not be distorted by too large error fluctuations, which are absorbed by the measured value formation into quanta, in other words by differentiating only coarsely. This coarse differentiation is now theoretically much easier to capture because the quantitative difference from a control group on such a coarse scale can be defined as the difference between the true means on that scale with a clear separation of the measurements. At best, the scale values concentrate at the extreme value in one direction with the least overlapping of the two distributions and the most powerful experimental manipulation of a theoretical construct. Of course, there are no error-free measurements but in our simulations (see Table 4) the standard variation is reduced from a continuous measurement to a quantized measurement from s_cont_ ≈ 1 to s_quant_ ≈ 0.04. The basic assumption behind this prediction is that the manipulation effect is the same for each subject and clearly observable. To use the mean of the scale as the parameter of the control group assumes that there is least restriction on the variability of the scale values under control conditions. Thus, if the scale can differentiate only three gradations, then these can be described as 1, 2, 3 and the control group should receive the value 2 as its mean, and the experimental group the mean value 3. The predicted difference is then *Δ* = 1. If one can distinguish 5 gradations, then one would set the mean of the control group to 3 and that of the experimental group to 5. Then the prediction would be Δ = 2. For an even finer measurement with 7 gradations, one would set the mean of the control group to 4 and that of the experimental group to 7. This gives a Δ = 3. For an even finer distinction with 11 gradations, the mean of the control group is normalized by 6 and the experimental group should theoretically get a mean of 11. The predicted difference is then Δ = 5. With these differentiations, we cover the reliabilities of r_XX’_ = 0.50, 0.80, 0.90, 0.95. A more accurate measurement of human responses should be possible only very rarely. Furthermore, experiments should be designed in such a way that they allow a clear differentiation to a control group. Then, depending on the reliability of the dependent variable, a point prediction Δ is possible. Furthermore, such differences are replicable, and they occur only extremely rarely by chance. In this way, one has a data basis for the construction of theories. These differences should be observed in many labs as a basis of a meta-analysis 3.0.

Although it is not possible to increase the expected value of the experimental group, it is already extreme, it is possible to decrease the value of the control group. In such a case, there is a possibility to achieve a larger difference Δ by the manipulation. Theoretically, this means that the originally chosen control group has already been influenced by the theoretical construct. This requires data which a meta-analysis 3.0 must provide when checking the control groups by elimination of the control subjects’ characteristics, if stable laws are to be formulated. This is the idea of moderators in a meta-analysis 2.0.

The task of a theory is more fundamental, not only to *describe* or *predict* effects in certain constellations, but also to *explain* these effects. These explanations are based on theoretical constructs as the cause of these effects which are not directly observable, also because they are supposed to hold for several empirical conditions. Through these theoretical constructs, empirical events are supposed to be attributed to a few common causes. Thus, the effects should not only remain stable and replicable for specific constellations, but different empirical constellations should be subsumed under a common theoretical construct. Thus, the effects must remain constant even over empirically different constellations, if they are represented as comparable in a theory. By a theory with its abstract constructs, one achieves the reduction of complexity to a few variables and regularities, from which then the observable effects can be predicted and explained. For this, one first needs stable effects in empirical conditions and must then try to explain these effects from a common theory. This always requires an abstraction. Without this stability in a common empirical or experimental condition, one cannot develop theories. An example is dissonance theory, which has obtained comparable effects under different experimental conditions and could be subsumed under a general theory of information integration (Witte, 1980; Witte and Heitkamp, 2006; Witte and Zenker, 2022). One can now go a step further and, for example, integrate within small group research such diverse approaches as social impact theory, the choice shift effect, minority influences, social decision scheme theory, social motivation, the structure of communication in small groups, self-categorization theory, and linking minority effects in experiments to innovation research in the field (Witte, 1987, 1994, 1996, 2005). Such theoretical integrations seem unconvincing at first. They cannot succeed at the level of empirical observations because direct empirical conditions are hardly comparable. It is only the quantitative reconstructions without fitting the data to the theory (over-fitting) and obtaining a good match in very different conditions that turns such integration efforts into serious theory constructions beyond pure speculation. One can also develop group facilitation techniques along this path of theory construction based on quantitative results (Witte, 2007; Witte, 2012). Finally, one can also relate small group behavior on evolutionary theory (Witte and Kahl, 2008). A prerequisite for theoretical connections is always data that show comparable effects. If a theoretical integration wants to stand out from speculation, it must be supported by data. Only then can it seriously claim scientific verification. Thus, a comparison between empirical results and theoretical assumptions is always needed. Such a comparison must not be based on pure induction and fitting a model to existing data. There must always be an abduction by the researcher’s view who translates the existing data into a theory from which predictions or explanations follow deductively. Thus, there always needs to be a standard of judgment for the similarity of a theoretical deduction and an empirical observation. We discuss the development of such a yardstick in the following point.

### 2.4 The similarity coefficient I_SIM_

We need independent tests of theoretical assumptions in different laboratories to gain some certainty about the quality of a theory. At the same time, we need guidance on how to modify a theory when there is a stronger divergence between data and theory. Such an index should be invariant to scale values and should not be based on a probability judgment between hypotheses, but solely on the similarity between the predicted theoretical measurement and the empirical result on a scale. We have developed such an index (Witte et al., 2022). It should be as transparent and easy to interpret as possible. Since ratios of differences are invariant with respect to an origin and a unit transformation, we chose the following expression:


$$I_{SIM}:\;=\;|\left(m_{\text{theo}}-m_{0}\right)|/|\left(m_{1}-m_{0}\right)|\;=ES_{\text{theo}}/ES_{emp}$$

$m_{\text{theo}}:\text{theoretical}\;\text{prediction}$


$$m_{0}:\text{mean}\;\text{value}\;\text{of}\;\text{the}\;\text{control}\;\text{group}$$

$$m_{1}:\text{mean}\;\text{value}\;\text{of}\;\text{the}\;\text{experimental}\;\text{group}$$

ES:effectsize

$$For|\left(m_{1}-m_{0}\right)|=0,\;I_{SIM}\;\text{is}\;\text{not}\;\text{defined}$$

For different signs in the numerator and denominator, I_SIM_ = 0.

If the prediction is correct, I_SIM_ = 1.

If we now look by simulations of I_SIM_ at the percentage of values that lie in a similarity interval of 0.80 to 1.20 when samples are drawn from continuous *t*-distributions with different mean differences and sample sizes, but which agree with the prediction, then we can estimate whether the prediction can agree with the observation. For already quantized data, the I_SIM_ values should all be close to 1 if the prediction matches the empirical data. With the variation of I_SIM_ one can see if the results are stable under replications in a specific context. One can also determine whether a global model leads to consistent predictions in different contexts when one varies the independent variables (Witte, 1996).

With this type of data analysis, one can set the stage for more global theory building, but this is driven by empirical data. Theory building must always be empirically anchored to avoid ending up in pure speculation. Quantitative reconstruction could also be an instrument in the methodology of psychology (Witte and Heitkamp, 2006). Furthermore, different laboratories must be involved in theory building to be able to identify questionable research methods and other shoddy approaches. Research in the empirical social sciences is a data-driven theory construction process involving collaboration among many research groups to combine the necessary resources. This collaboration must be guided by a common strategy. This can be the approach of meta-analysis 3.0 for an integration of effect sizes from different labs into a mean of quantized data as stable findings. Such effect sizes can be used by a methodology of quantitative reconstruction. But they should not be taken for a global statistical test of the effect. It is only a description of the similarity between data and theoretical prediction.

## 3 A discussion of the usual meta-analysis 2.0 as a tool for an estimation of an empirical effect for theory construction

As a starting point, the most recent nine examples of meta-analyses from the *Psychological Bulletin*, the central journal for meta-analyses, were selected as case studies, and a tenth due to some specific effects of temporal change. The methodological procedure will be considered and the gain in knowledge discussed, which is prefaced in a “Public Significance Statement” in almost each paper.

Following this individual case review, an (almost) representative selection from the published meta-analyses in psychology will be discussed in terms of their research contribution to the scientific development of the discipline. This will then lead to a conclusion about its importance for the discipline in general and its theoretical development in particular. The general theory deficit is emphasized again and again in our scientific community (Borsboom et al., 2021; Eronen and Bringmann, 2021; McPhetres et al., 2021; Muthukrishna and Henrich, 2019), and it is seen that this form of cooperation in meta-analyses 2.0 is not a way to counteract it. Since all empirical science relies on cooperation to test assumptions, because individual studies are not sufficiently trustworthy (Krefeld-Schwalb et al., 2018), one needs a method that can integrate multiple studies, as meta-analysis is intended to do. From the discussion of meta-analysis 2.0, consequences must be drawn that lead to the formation of meta-analysis 3.0, if one sees the main task of a science in theory construction (see also 2. above). In this regard, meta-analyses are generally not procedures that provide a combined significance test of a hypothesis, although this impression is often given. Hypothesis testing of integrated studies must be done in a different way, namely as likelihood aggregation. In meta-analyses, we take each case as only descriptive information about an empirically observed difference or an empirically observed association (correlation). Thus, the goal of a meta-analysis 3.0 must be to describe differences or correlations that remain stable and close to the true values in the population. These parameters should be as large as possible and homogeneous across studies, only then can one expect a common law behind the observed data at all, as discussed above. Furthermore, the observations should be consistent with theoretical expectations in order to accept the assumed regularity as generalizable. Meta-analyses prepared for theory development will have to proceed methodologically differently, but the principal idea remains: Multiple studies must always be used to obtain trustworthy results for an assertion of a theoretical prediction.

### 3.1 The variation of independent variables in current meta-analyses

When we look at the selected individual cases of meta-analyses 2.0 above, they examine common psychological assumptions in the independent variables that are intended to be supported in the dependent variables. In many cases, the meta-analyses support these everyday experiences on average, but with considerable variation. In fact, the statistical significance of the individual meta-analyses is whether our everyday beliefs are true on average. However, they vary considerably across contexts, given the data from each study in a meta-analysis. Thus, the goal of meta-analyses is to get an overview of whether and to what extent everyday psychological beliefs are true in different contexts. A random effects model is appropriate for this question (Hedges, 1983). The aim is not to find a certain stable theoretical effect in all studies, but to estimate the variation in different contexts and to assess the strength of the effect, i.e., how well do our everyday psychological ideas apply to the observed events. Thus, it is everyday psychology that is being tested, rather than an abstract theoretical construct that lies behind the everyday observation and can be generalized across studies as a fixed influence variable. Let us look more closely at 10 recent individual meta-analyses 2.0.

- Changing attitudes, intentions, or behaviors through messages is more effective when those messages are targeted to recipients, e.g., through the social similarity of the sender, the similarity of the sender’s behavior, or the sender’s membership in one’s group (Joyal-Desmarais et al., 2022). This result is summarized in the following “Public Significance Statement”:

“This meta-analysis of over 700 studies shows that persuasive messages are more effective when they are designed to match the motivational underpinnings of people’s beliefs and actions (e.g., their values or personality)”.

This result describes an everyday experience. The observed effects show considerable heterogeneity across contexts. The classification of the independent variables is based on everyday experience whether a communication is “made” or not. However, it is not explained why the observed effect occurs and in what way it therefore varies across contexts. In fact, it only describes that a certain effect occurs with a certain face-validated matching procedure.

- In another meta-analysis (Frankenbach et al., 2022), the difference between the strength of sex drive between the sexes is analyzed. Everyday psychology has developed ideas here and has identified a clear difference. The sex drive is stronger in men than in women. The independent variable as a classification is derived from the usual sex differentiation and the dependent variables are determined in the form of face-validity (sexual fantasies, sexual desire and masturbation). The result is described as follows in the Public Significance Statement:

“This article explains sex drive from a scientific, psychological perspective – operationalized as sexual thoughts, desire, and masturbation frequency – and provides support using a meta-analytic review that men have a stronger sex drive than women.”

- Some meta-analyses already refer to existing meta-analyses and try to summarize these results. In this case, Durlak et al. (2022) attempt to look at twelve meta-analyses together on the extent to which schools promote social and emotional learning. Thus, the focus is on a practical effect and not solely on confirming everyday ideas. The result is sobering from a theoretical point of view, because it does not reveal any evidence about the regularities, as summarized in the “Public Significance Statement”:

“This review of 12 meta-analyses, involving an estimated 1 million students from early childhood education through high school, shows that social and emotional learning (SEL) programs have consistent, positive impacts on a broad range of student outcomes including increased SEL skills, attitudes, prosocial behaviors, and academic achievement and decreased conduct problems and emotional distress. However, there is little consistency regarding conditions and mechanisms by which these programs are most effective.”

This effect is rather larger than usual effects and homogeneous across different contexts. This is an empirical confirmation of everyday assumptions and a justification of schooling, but not an explanation of such an effect.

- A fourth meta-analysis focuses on cognitive control in individuals diagnosed as depressed (Quigley et al., 2022). Depressed individuals are worse at controlling negative cognitions than healthy individuals, who in turn are worse at controlling positive cognitions. This is also consistent with an everyday psychological assumption. The Public Significance Statement summarizes the results as follows:

“This meta-analysis indicates that depression-vulnerable individuals, including individuals with current major depressive disorder, remitted MDD, and dysphoria, have impaired cognitive control of negative stimuli relative to neutral and positive stimuli.”

The reported effects are small, but they confirm everyday beliefs.

- In another meta-analysis on depression (Rueger et al., 2022), a small effect is found when parental support is considered. The nature of support as an independent variable is determined by a rating of the investigators and the limitations of the study point out that reliable and valid measurement instruments are lacking for this variable. The Public Significance Statement summarizes the results as follows:

“This meta-analysis demonstrated the importance of perceived social support from both mothers and fathers in relation to lower levels of depression in children and adolescents with modest youth gender differences.“

Thus, we again get confirmation of everyday beliefs without further theoretical explanation and without reference to the previous study on cognitive control in depression.

- A meta-analysis by Powers et al. (2022) examines adolescent decision-making behavior that relates to risk taking. A small effect is found for increases in risk-taking as a result of observation, but only when the observed person suspects that higher risk-taking is valued positively. The result is summarized as follows in the “Public Significance Statement”:

“The present meta-analytic review found that peer observation increased adolescents’ tendency to make risky decisions, but the effect is small in magnitude and was much greater when peers were expressing pro-risk preferences.”

One does find a small effect confirming the everyday assumption that adolescents in the group situation of an observation show themselves to be more willing to take risks, but only if this willingness to take risks is also evaluated positively by the peers. It is worth noting in this context that from early small group research this value-dependent change (choice shift effect) is known and explained by the normative pressure in the situation (see 7.2). However, such theoretical references are not made.

- Another form of meta-analytic research is to consider the results of meta-analyses together with a look at theoretical predictions. Here, the meta-analysis of Ryan et al. (2022) is such a special exception, because it looks from a global theory at individual partial theories together and to discover the regularities behind them. This is only possible to a limited extent because there is a large heterogeneity in the results and some of the effects are small. The partial theories are also not really integrated, only the many results can stimulate to construct a theory which is important in many practical areas. The result is summarized as follows in the “Public Significance Statement”:

“This systematic review includes a comprehensive narrative synthesis of 60 meta-analyses, each of which tests various principles form self-determination theory (SDT). Such a review is of broad public significance because SDT has become one of the most widely applied approaches to human motivation and is the basis for interventions in many domains including work and organizations, health care, education, physical activity, and sport, among others.”

Again, this paper is not about developing a theory, but about empirical effects in different domains to stimulate a theory construction that can address these differences.

- In a meta-analysis by Koutsoumpis et al. (2022), the relationship between linguistic categories and the classic Big Five personality dimensions is examined. For this, one looks at the correlations of linguistic categories with the measures on personality dimensions and selects the linguistic categories that show a significant correlation with the personality dimensions. This is a purely inductive strategy that looks at observed effects. These effects are small and heterogeneous. There is no notion of why which linguistic categories should correlate with the personality dimensions, but there is software that identifies these categories and can be easily used for data collection. The result is summarized as follows in the “Public Significance Statement”:

“This meta-analysis identifies the linguistic categories (i.e., word categories, such as negative affect) that individuals use depending on their personality traits, as well as the linguistic categories that other people use to draw personality inferences. Individuals indeed use specific linguistic categories depending on their personality traits and others use specific linguistic categories to draw personality inferences, but those relations are dependent on study and tasks characteristics (e.g., text length, Linguistic Inquiry and Word Count version).”

These small and heterogeneous effects do little to increase knowledge of psychological regularities, although it would have been nice to be able to diagnose personality this way.

- Another meta-analysis is about improving memory performance through actions. In this study by Roberts et al. (2022), a very large effect is reported. This also seems to be stable and can be practically used for memory performance. Patient samples (Alzheimer’s, Parkinson’s) are also used and computer tomographic results are integrated. The result is summarized as follows in the “Public Significance Statement”:

“The enactment effect is the finding that physically performing an action represented by a word or phrase leads to enhanced memory for that information relative to simply reading it. This review integrates evidence from behavioral, neuroimaging, and patient studies to highlight the utility of encoding multiple facets of an item or an event to enhance its retention. Enactment was found to be a reliable and effective mnemonic tool for both neurotypical and patient populations.”

This meta-analysis also tries to differentiate between two theoretical approaches based on the data. But this is not clearly decidable according to the data. However, such a meta-analysis could be a starting point for an abduction about memory having large effects in different contexts and different populations.

- To conclude with, the tenth randomly chosen meta-analysis 2.0 analyzes changes of effect sizes over time. The topic of Karazsia et al. (2017) will be considered as a well-known problem of people, namely, the satisfaction of individuals with their physical appearance whereby women are shown to be more dissatisfied than men when considering body weight, and men to be more dissatisfied with their muscle development. This difference seems to apply worldwide and leads to a large effect between the sexes. The result is presented as follows in the conclusion (p.307), because the “Public Significance Statement” is missing in this article:

“Our results revealed that neither thinness-oriented nor muscularity-oriented body dissatisfaction has changed significantly for men, and thinness-oriented body dissatisfaction has decreased for women across a 30-year period.”

The insight gained from this meta-analysis is not great beyond everyday psychological expectations except the decrease in the dissatisfaction for women.

If one looks at these individual cases, which were not selected due to content, but almost solely based on the timeliness of their publication in the *Psychological Bulletin*, then arbitrary effects can be selected as a subject of study. There is no systematicity of content and no connection between the results. Such a form of addition of arbitrary effects in the publications cannot advance a science (Witte, 2023). Now the general empirical results of meta-analyses 2.0 beyond single cases will be discussed in more detail.

### 3.2 Overview of the results of classic meta-analyses 2.0

The meta-analyses reported as examples cannot provide an overview of the general results also in other journals. There is now an attempt to produce a representative selection of published meta-analyses 2.0 and to look at their results in more detail (Linden and Hönekopp, 2021). Quoting the *abstract*, it is clear that theoretical development cannot be expected from the existing meta-analyses: “Typical levels of heterogeneity thus offer a useful but neglected perspective on the levels of understanding achieved in psychological science. Focusing on continuous outcome variables, we surveyed heterogeneity in 150 meta-analyses from cognitive, organizational, and social psychology and 57 multiple close replications. Heterogeneity proved to be very high in meta-analyses, with powerful moderators being conspicuously absent. Population effects in the average meta-analysis vary from small to very large for reasons that are typically not understood.”

Globally, the following configuration becomes apparent - small effects from a random hypothesis are homogeneous and larger effects are heterogeneous. With such a result, one cannot empirically develop theories as overarching regularities because one has no empirical basis for theory construction as an abduction process. As data in the meta-analyses, *d*-values, *g*-values, *r*-values or *z*-values are used. These values are considered as measurements of a sample from a population in which such an effect is generated by the independent variable. Parameter estimation procedures have become very sophisticated, and one mainly uses a random effects model to be able to generalize statistically to the population.^7^ Thus, one expects a relation between the meta-analytical results as a sample and the theoretical populations with diverse true effects to which one wants to generalize. To be able to establish this relation in the usual meta-analyses 2.0, as exemplified above, does not seem to us to be given. In fact, the observation of heterogeneity is sufficient to consider such a reference inappropriate. One can now ask why this heterogeneity occurs. It starts with the selection of the studies, which are chosen from a large database via word searches. The word meanings and methodological implementation in the studies can vary widely. For the independent variables, we are often dealing with everyday ideas and the dependent variables are rarely controlled for reliability and validity. The study context is not very standardized. This already leads to the fact that even a specific study can rarely be replicated (Baumeister et al., 2023; Nosek et al., 2022). Consequently, there are uncontrolled influences in the individual studies that will generate heterogeneity also due to the assumption that there are different distributions with different true effects under the random effects model. Furthermore, the parameters used in the meta-analyses under a fixed effects model are two-dimensional when considering the *d*-value with the mean difference weighted by the standard deviation: *d* = (*m*_1_-*m*_2_/*s*). Thus, there are two sources of variation in these *d*-values, resulting in variation in the *d*-values when there is greater variation in the standard deviation across studies, even though the mean difference may be constant. The problem of biased sampling from the analysis of published data is brought to the attention of many of us on several occasions because we are asked to provide unpublished but finished papers. This publication bias can be avoided, according to many, by allowing the study to be reviewed without results and then publishing non-significant results. It is important to note that the logic of our statistical reasoning is based on falsificationism, and only significant results are meaningful. All non-significant results do not support the null hypothesis according to this logic. For this, one needs a different test statistic (Witte and Zenker, 2017a; Krefeld-Schwalb et al., 2018). Since the effect measure is two-dimensional, such an insignificant result can also be generated by a high error variance. Therefore, according to this falsificationism in the usual testing logic, such insignificant results should not to published. Consequently, one will never be able to draw an unbiased sample from published results. However, if a sample does not have a clear relationship to a population, then it cannot be used to draw an inference about the population. If we want this inference, then we are dealing with a finite population from which we can draw at least an approximate random sample. What is possible, however, and meta-analyses are excellent for this, is that in a highly controlled condition, you measure the effects in multiple studies at different laboratories using the same measurement tools. These effects must be homogeneous and only the mean differences are considered. No generalization is made to any infinite theoretical population; only the observed effects are considered. However, by varying across laboratory conditions at high control, very similar effects should emerge. The source of variation in location, time, and measurements should not matter. Only then is the independent variable, e.g., an intervention, interpretable at the mean. The error variance around the mean should be small and comparable across all studies. Such a meta-analysis leads to a trustworthy estimate of effects, but it does not explain that effect. Behind this idea is the concept of evidence-based research in an applied context (Witte and Zenker, 2022).^8^ If meta-analyses 2.0 report small effects, provide heterogeneous data, are not suitable for generalization to a population, use arbitrary independent variables from an everyday psychology, and not validated dependent variables, then they can only describe the limitations of the findings but do not contribute to the *progress* of scientific psychology. Nevertheless, only results are trustworthy if they are replicated in individual studies and generalizable across variations in location, time, and measurements. One needs a meta-analysis 3.0 for guiding theoretical progress in knowledge by providing integrated parameter estimates not by induction but by abduction that can be the basis for integrating studies under generalized regularities, i.e., theories. There are specific requirements for the empirical basis that must be met by a meta-analysis 3.0 for an *abduction process* to be successful for the construction of theories.

## 4 A conception of a meta-analysis 3.0 for the construction of theories

In general, meta-analyses 3.0 are not intended as a basis for integrated theory *testing*, but solely as an ensemble of empirical results in different study conditions for a formulation of precise hypotheses as a *basis* for theory testing. An integrated testing of theories can – as mentioned – only be done via likelihood aggregation (Witte and Zenker, 2017b; Krefeld-Schwalb et al., 2018). However, this already requires a concrete point hypothesis or a concrete interval hypothesis with two points. One must derive these from empirical values without fitting them to data from samples (over-fitting). Unfortunately, one can test theories only if one has developed some. Just assuming a deviation from chance, even with a one-sided determination, is not yet a theory which can explain these empirical results. It follows logically that a process of specification must have preceded that allows for such testing. This specification process should be guided by empirical data but should not simply derive the theoretical assumptions from an inductive fitting process, because this fitting always implies an adjustment to error-prone data, which may yield incorrect parameters that deviate significantly in other samples due to the over-fitting problem. Thus, meta-analysis 3.0 is the crucial basis for the proposed abduction process to build theories (Gervais, 2021; Muthukrishna and Henrich, 2019; Smaldino, 2019).

Meta-analyses 3.0 will benefit from a research culture where the measured constructs rely on a theoretical foundation common to several researchers, which is not to be mistaken by a pre-planned multi-centered research agenda (multi-lab standardized replication). In fact, in order to obtain *generalizable* theoretical assumptions (in particular across settings) by abduction, a uniform setting or standardized experimental conditions are not even expected or desirable at all (conceptual replication). In turn, an adoption of this method may set a signal fostering such kind of coordination, which is the meaning of Witte and Zenker’s (2017a, p. 2) claim that “our community should come to coordinate itself on joint long-term research endeavors.”

### 4.1 The generation of measurements with small measurement errors

Every measurement is subject to error. Any measurement with human measurement subjects will have limited reliability. One can assume *a concept of anthropometric reliability* by looking at the reliabilities of various scales that have a mean reliability of r_TT_ = 0.80 or at best r_TT_ = 0.90, which leads to a measurement error for the observed values. This measurement error, in turn, depends on the measurement instrument, i.e., the scale used. If one wants to measure a theoretical variable, then one should achieve at least such an anthropometric reliability, otherwise one cannot expect stable results which can still differentiate sufficiently (Witte and Stanciu, 2023). As outlined in Chapter 2.2, on such a scale with r_TT_ = 0.80, only 5 levels can be reliably differentiated quantitatively. In a meta-analysis 3.0, only scales with the same reliability should be used, because otherwise the empirical results do not provide a stable indication of the theoretical population parameter. As an example, if one measures the dependent variable with a similar reliability of r_TT_ = 0.80, then one can conduct a meta-analysis 3.0 across several individual studies. If the scales used should even have a reliability of r_TT_ = 0.90, then one would be able to differentiate 7 quanta in a stable way. Thus, a meta-analysis 3.0 requires the control of the reliabilities of the dependent variables and must not integrate arbitrary measurement instruments with different reliabilities.^9^ The quantization of the measurement values leads to a very small measurement error, so that the observed and quantized mean values can be regarded as *de facto* equivalent to true values for the respective control and experimental groups (Witte and Stanciu, 2023; https://osf.io/9ap6m/). The difference in means should be assumed to be the true difference in the population in the studies.^10^ This empirical difference should also be consistent with the theoretical prediction. The extent of deviation between predicted and observed difference can be determined and an index can be used to evaluate this extent of deviation between theory and observation (Witte et al., 2022).

With this coarse differentiation between the mean values, it is also easier to make a theoretical forecast. It is not about fine gradations of measured values, because these are not reliable, but about quantitative categories (quanta) in which differentiation is made. The individual case on a scale is subordinated to a quantum (error interval) and the mean of these quanta is the empirical result of a psychological measurement of subjects on a theoretical construct. Consequently, it is about measuring theoretical constructs as a starting point and not about measuring arbitrary differences from everyday life or previous experiments. A meta-analysis 3.0 must already start with theoretical constructs and regularities before every day psychological effects are considered together with scales of low reliability and unclear validity. In fact, such an empirical observation has been made in a large number of meta-analyses 2.0 (Linden and Hönekopp, 2021). The advance in theoretical knowledge from these types of meta-analyses 2.0 is minimal. However, the *practical* benefit can be determined in highly controlled studies via a stable empirical effect without being able to explain it (Witte and Zenker, 2022; https://osf.io/zng8k). In general, this is the approach of evidence-based research. Here, the theoretical foundation is missing. This brings us back to the starting point of a meta-analysis 3.0. It is based on a theory. This theory is based on a scientific object definition of psychological phenomena (Witte, 2023) beyond the usual description “Psychology is the study of how people think, feel, and behave.” How can the crucial elements of psychology be specified scientifically without reducing it to everyday psychology? Here lies the starting point for a meta-analysis 3.0, but at the same time the starting point for the development of psychology as a science (Witte, 2023). The requirement for a meta-analysis 3.0 also emphasizes the role of theory-building of the research process. The effort required for a meta-analysis 3.0 is substantial and may result in the theoretical assumption not being acceptable when tested (via likelihood aggregation). Meta-analyses 2.0 always have a result in describing the empirical observations (see 3.1). However, without a tool like meta-analysis 3.0 there is no abduction process possible grounded on stable data. Without abduction there is no meta-analysis 3.0. This circular process is the center of the improvement of not only psychology (Witte, 2023).

### 4.2 A hypothetical problem for a meta-analysis 3.0 with some special problems as examples

Suppose we have the idea to clarify a theoretical question with the help of a meta-analysis 3.0, which has remained unanswered so far. Not only the procedure will be discussed as an example, but also the difference compared to meta-analyses 2.0 will be indicated together with some special problems. Furthermore, the kind of problem is different from the every-day effects under the meta-analysis 2.0.

The starting point in a meta-analysis 3.0 is always a theoretical question rather than an everyday assumption. For example, there has been intensive research on the issue of group polarization because it was originally assumed that small groups would always agree on their mean position of individual starting conditions after a discussion. However, an unexpected extremization occurred after a group discussion about some problems called choice dilemmas. This extremization as an effect is to be explained. Two causes for this change are assumed, first a pressure to adapt to the group norm (normative) and second a change in the content of the discussed problem (informational). This distinction is old (Deutsch and Gerard, 1955). However, it can be readily incorporated into a larger theory of small group processes (Witte, 1996). Further, there is a contrary claim that should be clarified. Vinokur and Burnstein (1974) suggest attitude change through arguments via the informational route, and (Witte and Arez, 1974; Witte and Lutz, 1982) cannot identify any cognitive change produced by new information, although there has been extremization. According to them, there should have been attitude change among group members via normative influence. But then, in the case of extremization, the mean of the individual positions in the small group before discussion will not be the convergence points, but ideally a point distinctly different, representing the social value of the problem. Roughly, these two sources of influence can also be described by means of an affective pathway (normative) and a cognitive pathway (informational).

For a meta-analysis 3.0 investigating a theoretically postulated influence process, a common understanding about the variables of interest would enable researchers to extract a common signal from studies by different authors, as described above. The independent variable differentiates between value-laden pro-or-contra-risk-and neutral problems. In choice-shift research, this is the mean of the individual ratings of the choice-shift problems on a risk scale. Here the first problem arises in validation because an outcome of 50:50 is not the neutral point in subjective risk assessment. It is higher, empirically at 63: 37. Only when the mean deviates from this neutral point, the social valence of the problem is clearly considered to be a problem of caution or risk character, resp. One can still capture the social value of problems by having them assessed via an instruction that asks for a judgment of what should be done in response to the choice shift problem. By this validation the independent variable is determined into neutral and valence problems pro risk or pro caution.

For the dependent variable, it is now a matter of assigning the individual arguments in the group discussion to the risk scale. One must now determine the reliability of these assignments to know the content of each argument on a risk scale from 1 in10 to 10 in10 (do not change the status quo for a positive alternative). Thus, one selects all the arguments that have been presented and has them rated on the risk scale by a larger sample of people like the subjects in the study group. Reliability in this case is the uniformity of the ratings across the sample of raters. This can be determined using a Q-factor analysis and taking the standardized variance of the first principal axis as the variance of the true assessments. Reliability is then the standardized eigenvalue of the first principal axis, or the measurement error s_E_, the square root of the standardized eigenvalue’s deviation from 1: s_E_ = √ [1-(standardized eigenvalue)]. The measured value for each argument on the scale should then be quantized to eliminate the measurement error. Each argument is then given the mean over the quantized measurements from the sample of raters. The theoretical prediction is that the mean of the individual ratings on a risk scale in the group will match the mean of the arguments put forward in the discussion (Witte and Lutz, 1982). This is true for value-based and neutral choice dilemmas. In terms of content, the arguments do not indicate a more extreme position on the risk-scale, although there is a change in the mean risk-values, according to the empirical observation. Thus, theoretically, it must be a normative influence if a change in means occurs in the group without substantive evidence of this more extreme evaluation in the arguments. Such a laborious test in several laboratories only makes sense if there is a real theoretical advance. This only occurs when this theoretical assumption is embedded in a larger theory as an element (Witte, 1996).

This example was also chosen because it involves two additional problems. If one formulates a theoretical assumption that predicts no difference between the independent variables (*m*_1_ = *m*_2_) then one cannot assess this value using the developed index I_SIM_ (Witte and Zenker, 2022) because there is a zero in the numerator. Then this index is always zero, however good the prediction is compared to the empirics. Thus, in this case, one must use a different quantification to assess the goodness of similarity between theory and empirical results. As a proposition one can use the percentage of groups whose quantized measured values lie in the error interval around zero as agreement with the theoretical forecast. One then has, for each small group, the individual pre-discussion risk levels and the ratings of the arguments on this risk scale. If one takes the risk levels to be stable and the quantized arguments as well, then the mean of the risk levels in the small group before the discussion should match the mean of the quantized arguments in the group. This is true for value-laden and neutral problems. People only present their individual position in the arguments. However, consensus on a common position occurs in the value-laden problems in a more extreme position than the mean of the initial levels, even though the content of the arguments does not support a more extreme position. For the neutral dilemmas, there is also no consensus beyond the mean and the arguments also reflect the initial levels. In both cases, the information exchange is the same. The extremization cannot be explained by the informational path. This is the theoretical assumption that can also be supported in a single study (Witte, 1979). The hidden social value in the dilemmas then leads to an adaptation to this general social orientation, in that through the exchange of value-laden arguments, which were already implicitly the basis for the individual position, this value-saturation has led to an increased certainty in the “correct” choice of the risk level. This leads to an accommodation to this value-laden direction, and thus to an extremization as the dissenters in the group adjust. The group consensus represents a process of convergence to this value-laden position without drawing on new arguments. Consequently, this extremization is a process of adaptation to a general social value (conformity) rather than cognitive restructuring (innovation) through new arguments.

A second problem is the statistical testing of the hypothesis when one expects a null difference. One cannot test this with a standard significance test, but one can determine the likelihood ratio for a hypothesis that assumes the null difference against a hypothesis that assumes a deviation from zero on the order of the measurement error: *d* = 0 versus *d* + *s*_E_. This deviation is the minimum qualitative jump in the dependent variable, when referring to quanta. Such a likelihood ratio can be calculated for each individual study in a meta-analysis 3.0 and these ratios aggregated over all studies. This aggregated likelihood ratio can then be tested against a critical value that depends on the total sample of pooled individual samples and relates to the measurement errors (*α*, 1-*β*) of this total sample (Krefeld-Schwalb et al., 2018). This is not possible when using the results of individual studies in the form of a combined significance test, although this is a common procedure in meta-analyses 2.0. This has already been shown in Birnbaum (1954). Meta-analysis 2.0 is not possible for a statistical test in the logic of the common significance test. Often, smallest effects become significant in very large aggregated samples. However, these results cannot be explained theoretically because the possibilities for such small effects are almost unlimited, and above that, these mean effects are still heterogeneous, which means that they could have been generated very differently. The mean effect tested significantly is not a representation of the processes in the individual studies.

Such an example illustrates the research effort required for a theoretical development, and at the same time, such an effort can only be justified if it is related to the development of larger theories. This sheds a critical light on the justifiability of meta-analyses 2.0: considerable resources are expended, and the epistemic value stagnates or is presented in textbooks as unjustifiably certain (Bartels, 2023).

The hypothetical path of an empirical science psychology can only be done through a form of meta-analysis 3.0. Meta-analysis 2.0 cannot promote this development because it distracts from the necessary path, apart from highly controlled studies in an evidence-based research.

### 4.3 Quantitative comparison of meta-analysis 2.0 with meta-analysis 3.0 based on 10,000 simulated studies in each meta-analysis

We want to limit ourselves to the *d*-value as the measured value in the meta-analyses 2.0 and take a closer look at this *d-*value to see what significance it has for theory formation. The theoretical statement for the distinction into two groups with the effect under consideration refers solely to the mean values. It was only recently realized that the observation of mean values has little to do with the individual measured values (McManus et al., 2023; Witte, 2024). It is therefore not possible to draw conclusions about individual reactions in the theory that uses the mean alone. If the individual reactions do not match the description of the mean difference, then the theoretical statement only applies to a minority of people. As described above, however, clear and stable mean differences in measured values that also apply to the individual characteristics are required for the theory construction. The more the measured values overlap between the control and experimental groups, the more blurred the experimental manipulation and thus the theoretically assumed effect becomes. At the same time, this effect should also be theoretically clearly predictable. Meta-analysis 2.0 can now be compared with meta-analysis 3.0 by expecting an exactly specified difference in the mean value depending on the reliability of the dependent variable and the sample size of the individual studies. If you use continuous measurements, then you must achieve a *d*-value of *d* = 3.3 to achieve this difference (see before; only 5% of values in the two groups overlap). If we now use quantized measured values, then a maximum difference in the quantization can be obtained much earlier. Demanding that on a quantized scale 95% of the individual measurements should follow the predicted maximal deviation from the control group as large, stable, and corresponding with the theoretical prediction, then we get the results in Table 5. Quantization therefore makes it possible to identify the existing discrimination in the reactions and to predict its extent on scales of different reliability for the purpose of theory formation by simulating studies with different sample sizes over 10,000 repetitions (Stanciu, 2022; Witte and Stanciu, 2023). The simulations followed *t*-distributions with mean values 0 and *d*, resp., and the appropriate degrees of freedom (Table 5).

**Table 5:** Resulting *d*-values in simulated data assuming 95% of experimental group show predicted maximum value.

Quanta	*N*	*d*	DIFF	percentage
3	20	1.1	1	0.95
3	30	1.1	1	0.98
3	50	1.0	1	0.98
3	100	0.90	1	0.97
3	999	0.80	1	0.998
5	20	1.5	2	0.94
5	30	1.3	2	0.95
5	50	1.2	2	0.95
5	100	1.2	2	0.99
5	999	1.1	2	1.00
7	20	No result	No result	No result
7	30	1.8	3	*0.92*
7	50	1.5	3	0.97
7	100	1.4	3	0.99
7	999	1.3	3	1.00
11	100	1.5	5	0.95
11	999	1.4	5	1.00
13	999	1.7	6	1.00

The first column describes the number of quanta. The second column describes the sample size of the individual tests. Column 3 indicates the average *d*-value for the continuous measurement, which results when at least 95% of the individual measured values can be observed at the maximum deviation from the measured value of the control group. The DIFF column shows the deviation from the measured value of the control group, which is always in the center of the scale. The fifth column shows the percentage of the individual measured values at this deviation DIFF. The percentage should be at least 95%. The percentage varies depending on the sample size. Slight deviations are marked in italics. If the percentage does not reach 0.95, this line is omitted (Table 5).

The table clearly shows that a demanded distinction between the measured values can be achieved through quantization rather than with continuous scale values. This is particularly evident when comparing the *d* values, which would have to be *d* = 3.3 for a continuous measurement if the two distributions only overlapped for 5% of the measured values. The simulation stopped increasing the *d*-values if 95% of the individual measurements reaching the maximal deviation in all samples together.

In the case of anthropometric reliability, this maximum difference can already be expected from a sample of N = 30, although the two mean values of the *t*-distribution only differ by *d* = 1.3 in the case of continuous measurement. If the *d*-value of the two continuous distributions is below *d* = 0.50, then no theorizing is possible because the individual measured values do not sufficiently follow the theoretical statement about the mean values. However, quantization enables a stable difference to be achieved much more quickly, which can also be used more easily for a forecast. The contemporary discussion about the conclusion from mean differences and the compatibility with the distributions of the individual values with the amount of their overlap has been criticized because psychology is person-centered and not average-centered (McManus et al., 2023; Witte, 2024). The mean difference must be consistent with the difference of the individual values or it is an average-to-person bias.

Obviously, the reduction of the error fluctuations still needs a *d*-value beyond the values found in the meta-analysis 2.0. It is not the small power of the object studies alone that causes the small empirical *d*-values in meta-analysis 2.0. We must look more deeply into the basic assumptions of an experimental study. The assumption is that a new information in the experiment changes the individual reaction likewise. However, this change is embedded into the whole experimental condition as a social situation that makes sense for all subjects in the control and experimental group. Control group and experimental group must be similar except the newly introduced manipulation which cannot be totally separated from the common interpretation of the social situation: The reaction in the experimental group is still a part of the global interpretation of the subjects. The main point is to decompose the sense-making elements in both conditions, i.e., what kinds of influence factors are introduced into the experimental group compared to the control group. These additional influence factors are the reason for a change of the individual reactions. They must be measured to predict theoretically the change of the means after the experimental manipulation. Such a change could be positive, negative or zero, and measuring these influence factors which quantifies the path of the influence from global interpretation in the control group to additional influence factors in the experimental group. The theory is based on the kind of combination rule and should explain this general rule. What is not explained in the experimental change is the mean of the control group as a combination of individual reactions. That must be based on decomposing the individual reasons for an interpretation before the experimental and control group started the experiment. This will be illustrated in Chapter 5.

## 5 Examples how to use the meta-analysis 3.0

In the following, we will give an actual example for theory construction with a meta-analysis 3.0. From the step in Table 2, data collection, abduction and formulating a theory, and using the fit coefficient I_SIM_ are involved.

### 5.1 Quantifying the choice-shift effect

In the past, there has been intensive research on the choice-shift effect, initially known as the risky-shift effect, a popular research topic in small group research (Witte, 1979, 1996). There a risk problem was to be discussed in a group and, if possible, a common risk and agree on a common risk level if possible. Surprisingly, the well-known result was an agreement that was more extreme than the mean value of the risk levels in the group, associated with all types of meta-analysis from 0.0, 1.0, and 2.0. In a comprehensive meta-analysis 2.0 (Barnir, 1998) on life dilemmas, there are tasks similar to the ones that we have used ourselves and with which we will conduct a meta-analysis 3.0 in the following. As a result of this meta-analysis 2.0, a large effect of *r* = 0.39 or *d* = 0.85 is reported according to Cohen’s classification. The variation of the effects across the choice dilemma problems is very large and one can only conclude that this effect exists under certain circumstances for certain problems. Nothing more can be inferred from this meta-analysis 2.0. The data do not provide a systematic approach for an abduction to discover a regularity. The empirical data correspond to the usual meta-analyses 2.0 and describe the empirical effects across 121 items in 14 studies.

In contrast, a meta-analysis 3.0 attempts to find stable mean values that can generate the empirical variation in the observed data via a theoretical integration. Abduction is only possible with trustworthy and stable parameters. This requires developing an idea of which stable background parameters are needed. It was clear after the initial research that each choice dilemma problem must be considered separately. Abductive consideration then led to the tentative assumption of three parameters for the individual orientation and one quantitative regularity. The next step was to provide stable estimates for these three parameters across different studies which is exactly the scope of meta-analysis 3.0^11^. Interestingly, it could be shown that one parameter can be eliminated because the occurring arguments are already determined by the other two parameters (Witte and Lutz, 1982, see point 4.2), i.e., the third summand in the following formula becomes zero.

The assumed regularity is expressed by the following formula describing the informational elements for an average subject in the experimental setting:


$$Y_{\text{theo}}\;=1\;SV+½\;\left(GS-SV\right)+1/3\;\left[AR-\left\{1\;SV+½\;\left(GS-SV\right)\right\}\right]$$

Y_theo_: individual value after the group discussion as a theoretical prediction from the corresponding mean values.

SV: social value as the assumed desirable risk level as mean value in a reference group (female, male, officer candidate). The estimation is computed via a sample from the reference group with corresponding instruction what risk level one should choose.

GS: small group standard as the mean value of the group members in the specific small group.

AR: the averaged risk values of the arguments in the group discussion rated by observers. This parameter could be eliminated because the difference between the arguments and the SV and the GS is zero.^12^


$$M_{\text{theo}}:\text{mean}\;\text{value}\;\text{of}\;\text{the}\;\text{individual}\;\text{prediction}\;\text{values}\;Y_{\text{theo}}\text{.}$$

$$M_{emp}:\text{mean}\;\text{value}\;\text{of}\;\text{the}\;\text{empirical}\;\text{reactions}\;\text{after}\;\text{discussion}$$

$$I_{SIM}:\;=M_{\text{theo}}/M_{emp}$$

The weights of the three components are chosen so that they also decrease with the decreasing amount of information shared and add up to 1. As a result, the values remain on the specified risk scale, and the hierarchical representation represents the degree of information sequence in this situation, from generally social in the reference group, via the concrete small group to the specific argumentation. We consider 3 studies, each with *N* = 50 subjects (female high school students, male high school students and officer candidates). These individuals discuss 13 risk problems in ten 5-person groups (Witte, 1979). The social value as a behavioral optimum has previously been collected from a different sample. I_SIM_ can now be directly computed as a fraction of the theoretical prediction divided by the empirical observation, because the scales are the same. This results in the values in Table 6.

**Table 6:** The I_SIM_ values for three samples with 13 choice dilemmas.

Choice dilemmas	1	2	3	4	5	6	7	8	9	10	11	12	13
Female	1.05	0.84	0.91	1.08	*1.27*	0.77	0.94	0.91	1.04	0.92	*1.46*	*1.28*	0.87
Male	1.10	1.09	0.97	1.04	*2.47*	0.93	0.88	1.02	1.05	1.11	*1.23*	1.05	0.96
Officer candidate	*1.26*	0.92	1.03	0.93	*1.47*	*1.23*	1.05	0.91	1.00	0.94	*1.34*	*1.27*	0.89

The means outside the similarity interval are italic.

If we consider the similarity interval between 0.80 and 1.20 as sufficient agreement (Witte and Zenker, 2022), then 11 forecasts out of 39 are not sufficiently like the theoretical forecast. That is 28% of all forecasts. In all three samples, the errors occur in problems 5 and 11 with an overestimation of the forecast compared to empirical data. If these two dilemma problems (which are the ones with particularly strong shifts toward the risky direction) are omitted, then there are 5 errors in 33 forecasts, which means a percentage of 15%.

If one takes a closer look at the mean changes for the sample of officer candidates (Hofstätter, 1986), one can see that those choice dilemma problems whose social value has a lower risk level than 0.63 and whose socially desirable evaluation before the discussion deviates in the direction of greater risk also change in the direction of risk, and those of the 13 problems that have a mean risk level before discussion above 0.63 and whose socially desirable value is also above 0.63 tend on average to be more cautious after the discussion. The correlation between the mean values before and after the discussion is 0.98. The regression line is:

M_after_ = −0.38 + 1.60 *M_before_ with M_after_ = M_before_ = 0.63 (Hofstätter, 1986, pp. 138–139). Without considering the influence components separately in the experimental manipulation, it is not possible to understand the observed mean value in the experiment. If one considers only the mean value across the 13 problems, as is usual in a meta-analysis 2.0, then the mean risk level remained almost the same M_before_ = 0.57 and M_after_ = 0.55. Therefore, the original theory needs to be modified: A quantification of the choice-shift effect needs to model a moderation effect as described. For a further, more detailed specification in a continued meta-analysis 3.0, more data and data reconstructions (from the points of view of the preliminary theories) are needed formulating a solid quantified theory about the assumed effect.

### 5.2 Reconstruction of classic experiments

Numeric similarities may support ideas, dissimilarities may disprove them or motivate refinements and possibly a deeper understanding. But similarities can also act as source for conjectures or conceptual abduction, e.g., regarding interconnections between different scientific topics.

The question whether the effects of Milgram’s (1974) classic experiments can be reinterpreted may serve as an example. The electric shocks are measured on a scale from 0 to 30. There are various experimental conditions that have a mean shock level of 22.5 (Witte, 1994). There is also a mean estimate by psychiatrists of when people will stop the experiment. If we now assume that the psychiatrists can record the participants’ assessment quite well on average, then this could be the socially acceptable reaction (compared to the SV above) that the participants can reconcile with their identity. The intervention of the experimenter urges participants to choose increasingly higher shock levels. He aims for shock level 30. If we now consider the psychiatrist’s judgment as a social value (SV) and evaluate the experimenter’s intervention as a default in the small group (GS), then the following law should result:


$$Y=1\;SV+½\;\left(GS-SV\right)=10+½\;\left(30-10\right)=20\text{.}$$

An average person should stop at level 20 in the experiment. If we now look at several studies, the mean shock levels are 27, 24, 21 and 18, and if we calculate I_SIM_, the results are: 0.74, 0.83, 0.95, 1.11. After all, three out of four theoretical mean predictions are inside the similarity interval of 0.80 to 1.20, providing support for the idea under consideration of a comparable integration rule of information.

Similarly, Asch’s classic experiment with the line comparison maybe re-interpreted in the same way. We assume 4-person groups with 3 confidents who uniformly name a false line (F) before the naive person responds. Then the prediction can be formalized:


$$\begin{array}{l} Y_{emp}=1\;SV+½\;\left(GS-SV\right)\;\text{with}\;SV\;as\;\text{the}\;\text{correct}\;\\ \left(\text{generally}\;\text{accepted}\right)\;\text{answer}\;\text{and} \end{array}$$

$$GS=¼\left(F+F+F+R\right)=¾\;F+¼\;R$$

$$Y_{emp}=R+½\;\left(¾\;F+¼\;R-R\right)=5/8\;R+3/8\;F=0.63\;R+0.37\;F\text{.}$$

Empirically, 36.8% errors were found. I_SIM_ is therefore 1 which would motivate a further consideration of the assumed regularity.

Many other reconstructions can be made and compared with the empirical data on I_SIM_. As a method, meta-analysis 3.0 provides parameter estimates across several studies and content areas that are based on the same regularities using informational elements and their aggregation.

What is not integrated into this research is the question why a choice dilemma is interpreted as a risk-prone, a caution-prone or a neutral dilemma from the view of a normative behavior in a reference group. Such a question must be answered by a cognitive representation of these dilemmas (Witte and Arez, 1974). The explanation of the small effect sizes by method effects (low power) alone is not the solution because also for almost true individual reactions the necessary effect sizes for an elimination of the average-to-person bias are beyond empirically found size effects. Psychology as a social science cannot separate the experimental influence from the individual interpretation. Meta-analysis 3.0 looks for a stable integration of informational elements into the reaction of an average individual in different studies beyond the automated literature search.

### 5.3 A detailed comparison of a meta-analysis 2.0 with meta-analysis 3.0

Initially, we outlined various forms of meta-analyses, the collection of results ranging from different object studies, ranging from meta-analysis 0.0 to meta-analysis 2.0. The further development of meta-analysis 2.0 to meta-analysis 3.0 can be linked to this development, because of the integration of object studies into a picture of empirical results. In 3.0, the individual studies are no longer regarded as a discovery because they report a deviation from chance, the random hypothesis. The quantitative results are no longer reported as observed average effects with corresponding significance tests, but the individual studies are measured by their agreement with a theoretical prediction. There is no statistical parameter estimation, but the individual studies should provide clear and stable results. These results are expected to be reproducible in different laboratories. In meta-analysis 3.0, we are therefore dealing with an intuitive procedure that does not use deviations from chance as a standard of judgment such as in meta-analysis 0.0, or as an average effect size of interventions in meta-analysis 1.0, or a combined statistical test of different studies in meta-analysis 2.0, but rather theoretical deduction with its stability and the size of the deviation from a control group. The mean values of the studies are considered and not parameter estimates from the data and the associated significance tests. It is about the stability of the information integration of the elements in the experimental group compared to the control group. If we consider the very comprehensive and systematic meta-analysis 2.0 by Quigley et al. (2022) as an example, then we can summarize that the meta-analysis for depressive patients^13^ registers it more difficult to control negative stimuli than neutral ones in 71 studies with 107 *g*-values, which correspond to the *d*-values, with a mean effect of *g* = 0.52. This value is significant at *p* = 0.001, but with *p* = 0.001 this mean effect is heterogeneous across the individual studies. The same applies to the control of positive stimuli: in 63 studies with 86 effects, the mean *g*-value is *g* = 0.30. This is also significant and heterogeneous at *p* = 0.001. Comparable results apply to neutral stimuli: In 45 studies with 61 effect measures, a *g*-value of g = 0.37 is determined, which is also heterogeneous. From this inductive approach of the meta-analysis 2.0, it can be concluded that a maximum of 33% of the individual measured values (*g* = 0.50) show a difficulty of control with negative stimuli. With the other two *g*-values of *g* = 0.30, approx. 21% show this difficulty with positive stimuli and with neutral stimuli (*g* = 0.37) it is approx. 25%. With this inductive approach and the heterogeneous results with only small effects, no explanation can be found, and no theory can be constructed. The average-to-person bias, the interpretation of the mean differences as differences on the individual level (McManus et al., 2023; Witte, 2024), is epistemologically not allowed if psychology is the science of single subjects. The first step will be to move away from computer-aided literature search, because nothing is said about the validity of the integrated studies’ measurements. Then we should look for stable integration processes over different studies and separate the studies with values of I_SIM_ inside the similarity interval and outside. For the studies inside we can try to theoretically explain the influence of the experimental manipulation by decomposing the information elements in the experiments and their integration rule. The heterogeneity is the result of the number and kind of the background variables in the experimental condition, their composition and the different integration rules. To find a stable effect size is unlikely. The empirical observation of the meta-analysis 2.0 is a natural consequence of the variable causes behind the mean of the single studies.

If we abandon the inductive method, we end up back at meta-analysis 0.0, which has chosen as a criterion the deviation from the meaningless random hypothesis as a scientifically significant discovery, but now in meta-analysis 3.0 the precise theoretical prediction on quantized scales in the individual studies is chosen as a scientifically significant criterion. In contrast to meta-analysis 2.0, the formulation of theories in 3.0 goes beyond evidence-based research by induction and remains a creative process guided by the intuition of individual researchers.

Both procedures integrate results from different perspectives, meta-analysis 2.0 with a focus on detecting heterogeneity or instability (random effects model as the statistical baseline (see Hedges, 1983) dispersion of individual values beyond the theoretical interpretation of the mean differences) in a specific topic, and meta-analysis 3.0 with the aim to detect stability in different fields of an average subject as a starting point for further consideration, i.e., abduction.

However, the meta-meta-analyses 2.0 are not designed for providing a consistent picture of the regularities in the data under a topic. Furthermore, an average-to-person bias can be recognized if the significant difference between two mean values is used to infer the difference between the individually measured values. Only with *d*-values of unattainable magnitudes of *d* = 3.3 does the substantive distinction between the mean values also apply to the distinction between the individual measured values in 95% of cases. Such an effect cannot be achieved in principle because an experimental manipulation cannot be decoupled from the control group to such an extent that such an effect could be produced. The control group and the experimental group must have a similar initial interpretation, only then there is an additional source of influence by the experimental manipulation. Experiments in psychology must measure an experimental effect on the basis of a common interpretation in both groups. Otherwise, the initial conditions differ, and no comparable results are obtained in the two groups. An additional basic assumption in the experiments is that the manipulation has the same effect on all subjects. No distinction is made between the different effects on the individual value of the participants. A higher degree of abstraction is chosen here in order to avoid having to investigate interaction effects with the individual position. Why this form of abstraction is not also chosen for measuring the individual assessments of the entire experimental and control condition seems to us to be scientifically inconsistent. Meta-analysis 2.0 then calculates two distributions of individual values. The *d*-measure is the extent of overlap between the two distributions in the control and experimental groups. It is not only the measure of the mean difference. A large effect and thus a small overlap of the two distributions is expected by a valid experimental manipulation. The less the two distributions overlap, the clearer is its effect. You can quantify the individual measured values if you know the reliability of the dependent variable on which the experiment is based. We then move on to a meta-analysis 2.0 but quantized values. However, the consideration of the individual values as a basis remains the same. Even under this condition, the d-values must assume an almost unattainable effect size so that mean differences and standard deviations of the individual measured values can be used consistently. These d-values describe the extent of the average-to-person bias and the impossibility of drawing conclusions about individual values from mean values differences with sizes usually observed.

When considering experimental effects as equal for each subject, this kind of abstraction should also be transferred to the whole situation and the data should be analyzed at the level of average persons the mean over subjects. Regularities can only be discovered if this abstraction step is also taken for the whole analysis. We are familiar with this form of abstraction in science when we compare cosmology and astronomy. Cosmology assumes for all laws that the universe is isotropic and homogeneous, i.e., the measures are averaged out on larger scales and the mean value can be used when forming theories. At the same time, it is clear from astronomy that this abstraction does not correspond to the observable universe. However, it is needed to form theories, but deviations can then be determined in individual cases.

A similar approach must be taken in psychology. The individual measured values are ignored and only the mean values are considered. These means are the subject of the theoretical considerations also in meta-analysis 2.0 and not the distribution of the individual values. If one now consistently refers to the mean values, then one would like to find the regularities of the mean value changes in the experiments and formulate them into regularities by abduction. Before one begins to explain supposedly found regularities by means through theories, one must check the claimed regularities in other laboratories, under other conditions, at other times (retrognosis), in other cultures etc. I_SIM_ was developed for this purpose. It evaluates the similarity between theoretical predictions and empirical results. This index describes the quality of the similarity between the forecast and the empirical result for an average individual. Meta-analysis 3.0 is designed precisely for this purpose. If quantized values are now used as measured values for the average individuals, then a meta-analysis 3.0 is already available with quantized values. However, this does not yet exist. Meta-analysis 3.0 can be used as an approximation, whereby in particular slight deviations from I_SIM_ beyond 0.80 and 1.20 can be checked with meta-analysis 3.1 by quantization of the single measurements on a scale.

If we now compare the two central parameters of meta-analysis 2.0 and 3.0, the d-measure and I_SIM_, the d-measure captures the mean value difference and transfers it to represent differences on individual measured values (the larger d, the more likely statements about mean value differences agree with the individual measured value differences), and I_SIM_ transfers the *similarity* of the theoretical prediction with the empirical observation of the average individual (the closer to 1, the better the similarity of prediction and empiricism). The single subject cannot be the element of theories because of all the error fluctuations beyond theoretical regularities.

## 6 Conclusion

If an essential aspect of science is theory construction as an increase of systematicity (Witte, 2023), then one must coordinate empirical research and develop a common, complex strategy. The idea of meta-analysis emerged precisely from this consideration but with other aims (applied interventions, experimental effects, observational effects). Further, research is a collaborative effort among groups. Moreover, research is always tied to empirical data if one wants to push back pure speculation. This must then lead to a strategy that methodologically guides the collaboration. Meta-analysis 3.0 is intended to be such a guide. It provides the foundation in the first place on which one can develop theories in a data-oriented manner. The theories developed in this way must then be tested inferentially, because there are always influences of error. The prerequisite for a theory test, however, is the creation of a theory beyond the simple discovery of significant deviations in the observations of single effects (Witte & Zenker, 2017a; Krefeld-Schwalb et al., 2018). Such a creation needs a tool like meta-analysis 3.0 for the necessary process of abduction, considering theory building as a data guided process grounded on stable and valid measurements with stable composition rules.

## Data Availability

The original contributions presented in the study are included in the article/supplementary material, further inquiries can be directed to the corresponding author.

## Footnotes

^1^In the more recent meta-analyses, statistical modifications are made to obtain parameter estimates with as little error as possible: Hedges’ correction for small samples, investigation of publication bias, and use of a random effects model compared to a fixed effects model. These corrections are based on the assumption of obtaining a better parameter estimate from the available sample of studies. For now, we consider only the basic approach.

^2^Such a registration is a kind of accounting of empirical observations like an economical inventory.

^3^This current intensive discussion in philosophy of science is presented in the two handbooks mentioned (Magnani, 2023; Magnani and Bertolotti, 2017).

^4^This citation points to the crucial point in meta-analysis 3.0. The rough deviation from the other (classical) methodological approaches for building and application of theories is presented in Table 1 to see the differences.

^5^The implication of this assumption is an odd number of categories if the number of categories should be symmetrical to the mean.

^6^Behind this idea are the following assumptions: If one could measure a psychological variable error-free, highly sensitive and in an uninfluenced state, each measured value carrier (person) would receive its own measured value. It can be further assumed that there are many different micro-influences acting on persons that change this original uniform micro-distribution into a macro-distribution - a sum of micro-elements - of a psychological variable to a normal distribution (Central limit theorem). Quantization now attempts to respond to the original maximum differentiation of the measurement carriers and the concrete influence observed on the measurement instrument. This is done via the construction of a measurement scale by keeping the number of *possible* measurement values in an interval constant (equally distributed - ergodic hypothesis) and adjusting the interval width to the reliability of the measurement instrument (see Figure 1).

^7^The basic assumption of the random effects model is given by Hedges (1983, 389):” Note that this model implies that there is no single true or population effect of the treatment across studies. Rather there is a distribution of true effects: Each treatment implementation has its own unique true effect.”

^8^That is the well-known approach to test a vaccine. A comparable approach in psychology is the many-lab test of a published effect in the literature. Such an effect should be stable and large to be trustworthy as evidence. The difference between a meta-analysis 2.0 and the many-lab test is the origin: the former is a fuzzy phenomenon of published data, the latter a highly controlled intervention. In both cases the expectation is a homogeneous and large effect, if the assumptions are true.

^9^Of course, it is possible to use the reliability of the scale with the least reliability as a conservative stable differentiation.

^10^This is the advantage of non-standardized measurements (Baguley, 2009).

^11^The use of the mean based on continuous data is an excellent approximation of the mean from quantized data, see Table 4. The main influence by quantization is the reduction of the standard deviation around the mean.

^12^Usually I_SIM_ is defined as a difference from a control group in the numerator and the denominator. This is necessary because the scale used has no meaningful origin. In this case and the two following cases the scale has a meaningful origin that is 10 (no risk), and in the Milgram experiment it is no shock on the shock scale and in the Asch experiment it is no false reaction. Thus, the measurements are on an absolute scale with a meaningful origin, so that measurements can be used without additional centration for measurements on an interval scale.

^13^This comparison is based on a parameter of the reference group and no experimental manipulation. The first step is to quantize the measurements to control error fluctuations. The reliability and validity of the measurements are not controlled so that the discovery of a classification effect has no comparison with a theoretical prediction. How can such an effect be used for building theories?
